# Ultraviolet radiation and the human eye

**DOI:** 10.5935/0004-2749.2021-0354

**Published:** 2023

**Authors:** Carlos Yuji Nunomura, Sidney Julio de Faria e Sousa

**Affiliations:** 1 Department of Ophthalmology, Hospital das Clínicas, Faculdade de Medicina de Ribeirão Preto, Universidade de São Paulo, Ribeirão Preto, SP, Brazil; 2 Faculdade de Medicina de Ribeirão Preto, Universidade de São Paulo, Ribeirão Preto, SP, Brazil

**Keywords:** Electromagnetic radiation, Ultraviolet rays, Eye burns, Ultraviolet filters, Visual disorders, Radiação eletromagnética, Raios ultravioleta, Queimaduras oculares, Filtros ultravioletas, Transtornos da visão

## Abstract

This work is a critical review of the current understanding of the effect of ultraviolet radiation on the eye. It deals with the classification of this radiation, environmental level, and the factors that determine it, along with penetration into the human eye, toxicity to ocular structures, associated morbidities, events that may increase the vulnerability of the eye, and artificial eye filters.

## Abstract (pt)

Esta é uma revisão crítica do efeito da radiação ul travioleta no olho. Trata da classificação dessa radiação, nível no meio ambiente e os fatores que o determinam, penetração no olho humano, toxicidade às estruturas dos oculares, morbidades associadas, eventos passíveis de aumentar a vulnerabilidade do olho e filtros oculares artificiais. Discute, ainda, o risco real dessas radiações ao olho humano à luz do conhecimento atual.

## INTRODUCTION

Electromagnetic radiation is a form of energy found in our environment that includes seven bands that are associated with attributes familiar to us, such as radiotherapy (gamma rays), radiography (X-rays), tanning (ultraviolet radiation), vision (visible light), heat (infrared radiation; IR), microwave oven (microwaves), and radio (radio waves). Typically, low-energy radiations, such as radio waves, are expressed in frequency (cycles per second), while high-energy radiations, such as ultraviolet radiation, are conveyed as wavelength in nanometers (nm). The relationship between frequency and wavelength can be demonstrated as follows:

$$v=\frac{c}{\lambda }$$

Where, *v* is the frequency, *c* is the speed of light, and λ is the wavelength.

Electromagnetic radiation behaves either as a wave or as a stream of photons. The first behavior is adequate for the study of energy transport, whereas the second is suitable for analyzing the light interaction with the matter, making it easier to understand the toxicity of wavelengths. The expression that relates the photon energy to the wave characteristics can be depicted as follows:

$$E=hv=h\frac{c}{\lambda }$$

Where, *E* is the photon energy expressed in ergs, *hv* is Plank’s constant, *c* is the speed of light, *v* is the frequency, and λ is the wavelength. Based on this expression, the higher the frequency and shorter the wavelength, the higher the energy of the radiation. That fact explains why ultraviolet light has more energy than IR.

**Figure 1:**
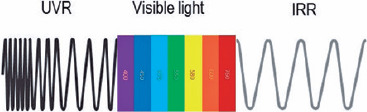
Sunlight spectrum. UVR, ultraviolet radiation; IRR, infrared radiation

Of the entire electromagnetic spectrum, solar radiation, despite being only a tiny fraction of it, is the one that interacts most with our ecosystem. It comprises three groups of wavelengths: ultraviolet radiation (100-400 nm), visible light (400-760 nm), and IR (760-10,000 nm). Under normal conditions, the human eye detects only visible light (Figure 1). The terms *ultra* and *infra* refer to frequencies and not wavelengths.

### Classification of ultraviolet radiation

Ultraviolet radiation was classified into three subgroups at the Second International Light Congress in Copenhagen in 1932^(1)^ (Table 1).

**Table 1:** Classification of ultraviolet radiation

UV radiation	Abbrev.	Wavelength (nm)	Features
C	UVC	100-280	Intense bioactivity. Completely absorbed by the atmosphere and does not exist naturally on Earth’s surface. Emitted by electric welding^(2)^, germicidal lamps^(3)^, and certain excimer lasers^(4)^.
B	UVB	280-315	Strong bioactivity. Inducer of Vitamin D3 in adipose tissue^(5)^. Responsible for tanning the skin^(6)^. Generated by high temperatures^(7)^, lamps for tanning and treatment of psoriasis^(8)^ and vitiligo^(9)^. Corresponds to 1/7 of ambient UV radiation.
A	UVA	315-400	Low bioactivity. Insignificant atmospheric absorption. Corresponds to 6/7 of ambient UV radiation.

UV= ultraviolet.

### Levels of ultraviolet radiation in the environment

Before attenuation by the atmosphere, solar radiation is composed of 52.8% IR, 38.9% visible light, 6.3% ul traviolet A (UVA), 1.5% ultraviolet B (UVB), and 0.5% ultraviolet C (UVC). The ozone layer eliminates all UVC before it reaches the Earth’s surface by preferentially absorbing the shorter wavelengths^(10)^. Since the upper limit of the stratosphere is about 50 km high, it is estimated that this radiation reaches the Earth’s surface with 3/5 of its initial value. As the atmosphere does not filter UVA significantly, it reaches our environment almost in its totality. Therefore, our ecosystem’s protection from UV radiation results from its absorption by the ozone column in its path.

The factors that influence the levels of UV radiation at the Earth’s surface comprise^(11)^ the following:

*Ozone layer* - the stratosphere’s ozone layer absorbs UV radiation in inverse proportion to its wavelength. Its protection varies across the day and year.

*Altitude* - UVB increases about 10% for every 1 km of altitude owing to the atmosphere’s rarefaction.

*Latitude* - the closer to the equator, the higher the level of UV radiation.

*Sun elevation* - the higher the sun is in the sky, the greater is the amount of UV radiation striking the Earth. Therefore, UV level varies with time of day and the time of year. The highest levels outside the tropics occur when the sun is at its peak in the summers.

*Clouds* - UV radiation levels are the lowest with cloudy skies.

*Reflection on the ground* - fresh snow reflects up to 80%, dry beach sand about 15%, and seafoam about 25% of the UV radiation.

### Penetration of electromagnetic radiation into the eye

The cornea and crystalline lens are the transparent media in the eye that absorbs the most UV radiation. The cornea filters out all UV radiation <300 nm. The crystalline lens absorbs all UV radiation <390 nm^(12,13)^. In other words, the retina is safe from this radiation under usual sun exposure conditions. Table 2 shows the total transmittances of the cornea and crystalline lens, sourced from the curves of Boettner and Wolter^(12)^. Regarding visible light, about 80% of it reaches the retina.

**Table 2:** Transmittance of ultraviolet radiation through the cornea and lens

UV	Wavelength (nm)	Transmittance (%)	
		Cornea	Crystalline lens
B	<300	0	0
B	315	40	0
A	320	60	0
A	380	80	0
A	390	80	10
A	400	80	15

UV= ultraviolet.

**Figure 2:**
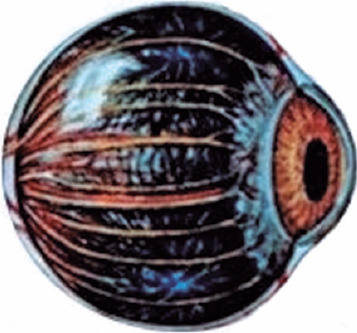
Choroid - the eye’s heat sink.

IR transmittance drops rapidly from 70% to 35% in the range between 700 and 1000 nm and even more sharply up to 1400 nm. The aqueous humor and the vitreous body play a significant role in this absorption curve^(12)^. Besides the natural protection of transparent media, the eye defends itself from these radiations through the choroid. It is a richly vascularized tissue separated from the neurosensory retina by the pigment epithelium (RPE), filled with black melanin pigments (Figure 2). The IR radiation absorbed by the cones and rods turns into heat that reaches the choroid via RPE. The choroid works as a heat sink (radiator) owing to the vast amount of blood circulating in its vessels. This mechanism is sufficient to prevent solar radiation from burning the retina as long as the pupillary diameter is ≤3 mm^(14,15)^. Other pigments such as zeaxanthin, lutein, and meso-zeaxanthin confer extra protection to the macular region via a similar mechanism. Because they have an absorption peak at 460 nm, they absorb about 40% of the blue light, whose energy is also relatively high^(16)^. The transmittance of IR radiation is relevant considering the suspicion that increased tissue temperature potentiates UV radiation and visible light toxicity^(14)^.

In addition to all this protection, the “reflex reaction to light glare” is manifested by pupillary constriction and contraction of the orbicular muscles of the face, with the consequent depression of the eyebrows, elevation of the malar regions, and reduction of the palpebral fissure, which together contributes significantly to the reduction of ocular exposure to all solar radiation.

### Eye toxicity of ultraviolet radiation

Absorption has precedence over transmittance and reflectance for electromagnetic interactions with biological materials. UV radiation absorption can either build or break bonds between atoms and molecules. Four factors influence both these events: *irradiance* — the number of incident photons per tissue area — measured in watts per square meter; *wavelength*, which is inversely proportional to the photon’s energy; *vulnerability* of the tissues to radiation; and the *exposure time*. Accordingly, we multiplied its irradiance by toxicity to determine the most destructive UV wavelength to specific tissues. Concerning the sunlight damage to the skin, for example, toxicity is measured in *Diffey* units, and the most destructive wavelength comes to 305 nm.

The damage of electromagnetic radiation to the biological tissues can be of three types: photothermal, photomechanical, and photochemical damage. The first injury results from the exaggerated temperature elevation at the cellular and molecular level, which causes denaturation of proteins, loss of tertiary molecular structure, and fluidification of the membranes. It corresponds to the “burn” in the conventional language^(17,18,19)^. The second is related to mechanical damage (micro cavitation) resulting from the sudden compression and decompression of the tissues generated by the exposure to extremely high amounts of energy, in a small area, for picoseconds. The typical example is that of the Nd:Yag laser. In the third type of damage, the energy absorbed by the tissue results in the breaking of the chemical bonds of molecules and the release of free radicals that, once generated, attack other molecules in a chain reaction^(20)^. This is the overall type of damage expected from UV radiation^(21)^.

One of the most important sites of ultraviolet radiation toxicity is cellular DNA^(22)^. Although UVB constitutes <1% of total solar energy, it can break one or both DNA helices or generate free radicals and oxidizing substances that damage it indirectly. UVA is less toxic than UVB because native DNA does not absorb it. However, it might induce photochemical damage via free radicals in situations of tissue vulnerability. Consequently, UV radiation exposure has been associated epidemiologically with a few eye conditions (Table 3). This table includes only those morbidities whose association with UV radiation is best supported by the literature.

**Table 3:** Morbidities possibly influenced by ultraviolet radiation

Morbidity	Site	UV
Welder’s keratoconjunctivitis^(23,24)^	Conjunctiva and cornea	B e C
Snow keratoconjunctivitis^(23,24)^	Conjunctiva and Cornea	B
Squamous cell carcinoma^(25)^	Eyelids	B
Squamous cell carcinoma^(26)^	Conjunctiva and cornea	B
Spheroidal degeneration^(27,28)^	Cornea	A e B
Pterygium^(28)^	Conjunctiva and cornea	A e B
Cortical cataract^(29)^	Lens	B

UV= ultraviolet.

**Figure 3:**
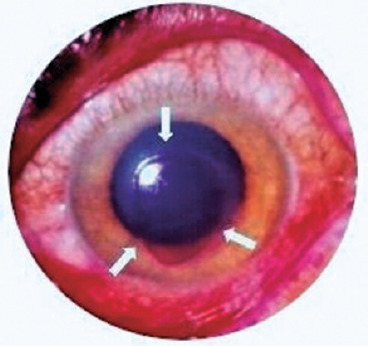
Arch-welding keratoconjunctivitis.

Of the morbidities in table 3, welder’s and snow keratoconjunctivitis occur from acute exposure to UV radiation. People who accidentally stare at the light from an electric welding instrument receive huge amounts of UVC and UVB. Snow skiers also expose themselves to high amounts of UVB, which is reflected from the snow-covered ground. These wavelengths impregnate the corneal epithelium, which is rich in DNA due to its high proliferation rate. About 6-8 h after the exposure, the epithelium dies and desquamates, leaving an extensive erosion, with severe pain and low vision (Figure 3). Fortunately, the phenomenon disappears spontaneously within 12 h. All other diseases mentioned in Table 3 require chronic exposure, genetic susceptibility, predisposing habits, and specific environments to become significantly influenced by exposure to UV radiation.

Since UVA has low bioactivity, we might expect damaging effects only in particular conditions of tissue vulnerability, overexposure, insufficient protection, or deficient repair of the irradiated tissues. Table 4 depicts these conditions in detail.

**Table 4:** Events likely to increase ocular vulnerability to ultraviolet radiation

Event	Predisposing factor
Acute overexposure	Work with electric welding, snow activities, direct observation of the sun.
Chronic exposure	Lifesavers, firefighters, ski instructors, agricultural workers, fishers.
Use of photosensitizing agents	Psoralens, phenothiazines, tetracyclines, sulfonamides^(30)^.
Genetic conditions	Albinism^(31)^, Retinitis pigmentosa^(32)^.
Artificial conditions	Aphakia (Lens absence).

### Artificial UV flters for the eye

Protection from UV radiation in sunglasses or colorless lenses results from UV absorbing substances used either in the form of the optical material or as a coating onto their surfaces^(33)^. However, polycarbonate materials, which naturally filter UV radiation, and pho-tochromatic lenses, which consume this radiation in the darkening process, do not need these substances.

An important concept is that the UV protection of sunglasses fits only the ordinary conditions of use. The wearer must not stare at the sun for more than 60 s, irrespective of whether the day is sunny, cloudy, or under a solar eclipse^(34)^. This value exceeds the absorbing capacity of these lenses. Professional use and solar eclipsis observation demand optical aids with higher absorption capacity in the UV, IR, and visible radiation spectrum.

Sunglasses must fully filter wavelengths between 300 and 400 nm to protect the ocular surface and crystalline lens. Lenses with absorption up to 380 nm transmit 40% of the ambient UV^(35)^. As UV protection generally comes with dark sunglass lenses, which inhibit the reflex reaction to light glare, the question arises whether these incomplete filters are worse than wearing no sunglasses at all.

Under conditions of increased vulnerability to radiation toxicity (Table 4), there is a trend to extend protection to 500 nm. The goal here is to eliminate blue radiation under the suspicion that it can damage the human retina similarly to that in laboratory mice^(36)^.

In summary, although UV radiation has a potentially toxic effect on the human eye, the risk and intensity of damage remain to be determined for the standard conditions of life on the Earth’s surface. Under the usual sun exposure, the human retina seems to be very well protected from this source of radiation, without causing any significant damage to the structures that perform this protection, that are, the cornea and crystalline lens. We must exercise great caution with inferences drawn from the laboratory animals, as the threshold for photic injury to the eye is highly variable between species, probably being the highest in humans^(37,38)^.

## References

[1] Diffey BL (2002). Sources and measurement of ultraviolet radiation. Methods.

[2] World Health Organization (WHO), World Meteorologcal Organization, United Enviroment Programme IC on N-IRP (2002). Global Solar UV Index: A practical guide.

[3] Takahashi J, Nakashima H, Fujii N, Okuno T (2020). Comprehensive analysis of hazard of ultraviolet radiation emitted during arc
welding of cast iron. J Occup Health.

[4] Kim SJ, Kim DK, Kang DH (2016). Using UVC light-emitting diodes at wavelengths of 266 to 279 nanometers to
inactivate foodborne pathogens and pasteurize sliced cheese. Appl Environ Microbiol.

[5] Fantes FE, Waring GO (1989). Effect of excimer laser radiant exposure on uniformity of ablated corneal
surface. Lasers Surg Med.

[6] Wacker M, Holick MF (2013). A global perspective for health. Dermato-endocrinol.

[7] Tadokoro T, Yamaguchi Y, Batzer J, Coelho SG, Zmudzka BZ, Miller SA (2005). Mechanisms of skin tanning in different racial/ethnic groups in response to
ultraviolet radiation. J Invest Dermatol.

[8] Oriowo OM, Chou BP, Cullen AP (2000). Eye exposure to optical radiation in the glassblowing industry: An investigation
in Southern Ontario. Can J Public Health.

[9] Toledo-Pastrana T, García-Hernández MJ, Carrizosa-Esquivel AM, Camacho-Martínez FM (2015). Evaluation of 25 years of phototherapy for treating psoriasis at a teaching
hospital in southern Spain. An Bras Dermatol.

[10] Hamzavi IH, Lim HW, Syed ZU (2012). Ultraviolet-based therapy for vitiligo: What’s new. Indian J Dermatol Venereol Leprol.

[11] Frederick JE, Snell HE, Haywood EK (1989). Solar ultraviolet radiation at the earth’s surface. Photochem Photobiol.

[12] Boettner EA (1962). Transmission of the ocular media. Invest Ophthalmol Vis Sci.

[13] Artigas JM, Felipe A, Navea A, Fandiño A, Artigas C (2012). Spectral transmission of the human crystalline lens in adult and elderly
persons: Color and total transmission of visible light. Investig Ophthalmol Vis Sci.

[14] Hope-Ross MW, Mahon GJ, Gardiner TA, Archer DB (1993). Ultrastructural findings in solar retinopathy. Eye.

[15] White TJ, Mainster MA, Wilson PW, Tips JH (1971). Chorioretinal temperature increases from solar observation. Bull Math Biophys.

[16] Loane E, Kelliher C, Beatty S, Nolan JM (2008). The rationale and evidence base for a protective role of macular pigment in
age-related macu-lopathy. Br J Ophthalmol.

[17] Birngruber R, Gabel VP, Hillenkamp F (1983). Experimental studies of laser thermal retinal injury. Health Phys.

[18] Birngruber R, Hillenkamp F, Gabel VP (1985). Theoretical investigations of laser thermal retinal injury. Health Phys.

[19] Henriques FC (1947). Studies of thermal injury; the predictability and the significance of thermally
induced rate processes leading to irreversible epidermal injury. Arch Pathol (Chic).

[20] Foote CS (1968). Mechanisms of photosensitized oxidation. There are several different types of
photosensitized oxidation which may be important in biological systems. Science.

[21] Jacques SL (1992). Laser-tissue interactions: Photochemical, photothermal, and
photomechanical. Surg Clin North Am.

[22] Sinha RP, Häder DP (2002). UV-induced DNA damage and repair: A review. Photochem Photobiol Sci.

[23] Podskochy A, Gan L, Fagerholm P (2000). Apoptosis in UV-exposed rabbit corneas. Cornea.

[24] Oliva MS, Taylor H (2005). Ultraviolet radiation and the eye. Int Ophthalmol Clin.

[25] Gallagher RP, Hill GB, Bajdik CD, Coldman AJ, Fincham S, Mclean DI (2015). Sunlight exposure, pigmentation factors, and risk of nonmelanocytic skin
cancer. Arch Dermatol.

[26] Sun EC, Fears TR, Goedert JJ (1997). Epidemiology of squamous cell conjunctival cancer. Cancer Epidemiol Biomarkers Prev.

[27] Johnson GJ (1981). Aetiology of spheroidal degeneration of the cornea in Labrador. Br J Ophthalmol.

[28] Taylor HR, West SK, Rosenthal FS, Munoz B, Newland HS, Emmett EA (1989). Corneal changes associated with chronic ultraviolet radiation. Arch Ophthalmol.

[29] McCarty CA, Nanjan MB, Taylor HR (2000). Attributable risk estimates for cataract to prioritize medical and public health
action. Investig Ophthalmol Vis Sci.

[30] Harber LC, Baer RL (1972). Pathogenic mechanisms of drug-induced photosensitivity. J Invest Dermatol.

[31] Rapp LM, Williams TP (1980). The role of ocular pigmentation in protecting against retinal light
damage. Vision Res.

[32] Heckenlively JR, Rodriguez JA, Daiger SP (1991). Autosomal dominant sectoral retinitis pigmentosa: Two families with transversion
mutation in codon 23 of rhodopsin. Arch Ophthalmol.

[33] Rifai K, Hornauer M, Buechinger R, Schoen R, Barraza-Bernal M, Habtegiorgis S (2018). Efficiency of ocular UV protection by clear lenses. Biomed Opt Express.

[34] Sliney DH (2001). Photoprotection of the eye - UV radiation and sunglasses. J Photochem Photobiol B Biol.

[35] Lappe C (2021). Protection against harmful uv radiation, even with clear eyeglass lenses.

[36] Grimm C, Wenzel A, Williams TP, Rol PO, Hafezi F, Remé CE (2001). Rhodopsin-mediated blue-light damage to the rat retina: Effect of photoreversal
of bleaching. Investig Ophthalmol Vis Sci.

[37] Różanowska M, Sarna T (2005). Light-induced damage to the retina: role of rhodopsin chromophore
revisited. Photochem Photobiol.

[38] Parver LM, Auker CR, Fine BS (1983). Observations on monkey eyes exposed to light from an operating
microscope. Ophthalmology.

